# Clinical and genotypic characteristics of 19 children with STXBP1-encephalopathy

**DOI:** 10.1097/MD.0000000000047269

**Published:** 2026-01-16

**Authors:** Lina Qi, Chengchao Fang, Zhonghua Hu

**Affiliations:** a Department of Pediatrics, The First People’s Hospital of Linping District, Hangzhou, China.

**Keywords:** children, epilepsy, levetiracetam, pathogenic variants, STXBP1-encephalopathy

## Abstract

To systematically summarize the clinical phenotypes, treatment responses, prognosis, and genetic characteristics of STXBP1-encephalopathy in Chinese pediatric patients, and to explore the clinical value of genetic testing in this disease. We retrospectively analyzed the clinical data, gene variant information, and treatment outcomes of 19 children with STXBP1-encephalopathy admitted to the Department of Pediatrics, Second Affiliated Hospital of Zhejiang University, between January 2020 and January 2024. Whole-exome sequencing was performed for genetic diagnosis, and Sanger sequencing was used to verify variants and confirm their parental origin. Pathogenicity of variants was classified according to the American College of Medical Genetics and Genomics guidelines. STXBP1-encephalopathy showed early onset, with 15 cases (78.9%) occurring within the 1st month of life. Five patients were diagnosed with Ohtahara syndrome, 5 with West syndrome, and 9 with unclassifiable early-onset epileptic encephalopathy. All patients had abnormal electro-encephalogram findings, mainly burst suppression (68.4%) and hypsarrhythmia (63.2%). Among the 19 patients, 1 achieved seizure freedom and discontinued antiepileptic drugs, and 4 achieved seizure control with levetiracetam. A total of 18 de novo pathogenic/likely pathogenic variants in STXBP1 were identified, including 7 novel variants: c.326-3(IVS5)delC, c.656del(p.Met219Argfs*13), c.746-747del(p.F249fs*6), c.798T > G(p.Y266*), c.1155delC(p.D385fs), c.1249G > A(p.G417S), and c.1250-2A > G. STXBP1 pathogenic variants are an important cause of early-onset developmental epileptic encephalopathy in Chinese children. Genetic testing is crucial for early diagnosis of STXBP1-encephalopathy. Levetiracetam shows good efficacy in controlling seizures in these patients, and early use is recommended. The 7 novel variants identified in this study expand the STXBP1 mutation spectrum.

## 1. Introduction

Epileptic encephalopathy is a severe neurological disorder in children, often lacking effective treatments or clear therapeutic options.^[1]^ Currently, advancements in molecular genetics and genetic testing have revealed that various mutations in different genes can cause developmental epileptic encephalopathy. This knowledge aids in early diagnosis and fosters research into its underlying causes.

STXBP1 mutations are linked to severe early-onset epileptic encephalopathies, including Ohtahara syndrome, West syndrome, and Dravet syndrome, as well as non-syndromic epilepsies, atypical Rett syndrome, and severe intellectual disabilities that do not involve epilepsy.^[2–4]^ These severe and often fatal infantile encephalopathies are marked by significant intellectual disabilities and cerebral dysfunction, typically accompanied by persistent epileptic activity, which ultimately results in the deterioration of cognitive, sensory, and motor functions.^[5,6]^ Although these disorders vary in their age of onset, developmental outcome, etiology, and seizure type, a common feature is that seizures are refractory to standard antiepileptic drugs, similar to most neonatal seizure disorders^.[7]^ Consequently, the prognosis is very poor.

In 2017, the International League Against Epilepsy (ILAE) identified a series of manifestations caused by this gene mutation, such as epilepsy and developmental delay, as STXBP1-encephalopathy.^[8]^ We conducted a retrospective analysis of genetic test results and clinical characteristics from 19 cases of STXBP1-encephalopathy. This study aims to improve clinical diagnosis and treatment of the disease while expanding the associated genetic profile.

## 2. Materials and methods

### 2.1. Study population

This retrospective study included pediatric patients (≤18 years old) diagnosed with STXBP1-encephalopathy at the Department of Pediatrics, Second Affiliated Hospital of Zhejiang University, between January 2020 and January 2024. Inclusion criteria: clinical manifestations consistent with developmental epileptic encephalopathy, including early-onset seizures (onset within 3 months of birth), obvious developmental delay or regression, and no improvement with conventional antiepileptic drugs; STXBP1 gene pathogenic/likely pathogenic variants identified by whole-exome sequencing and confirmed by Sanger sequencing; complete clinical data, including birth history, family history, seizure details, developmental assessment, medication history, and results of auxiliary examinations (electro-encephalogram, MRI, etc); follow-up duration ≥12 months. Exclusion criteria: epilepsy caused by clear acquired factors, such as intracranial infection, hypoxic-ischemic encephalopathy, and traumatic brain injury; comorbidity with other monogenic diseases or chromosomal abnormalities confirmed by genetic testing; incomplete clinical data or loss to follow-up.

Case ascertainment procedures: we 1st screened patients diagnosed with “developmental epileptic encephalopathy” or “early-onset refractory epilepsy” from the hospital’s electronic medical record system. We then collected the genetic testing reports of these patients and selected those with STXBP1 gene variants. Sanger sequencing was performed to verify the variants, and parental testing was conducted to confirm de novo origin. Finally, the diagnosis of STXBP1-encephalopathy was confirmed by combining clinical manifestations and genetic results.

### 2.2. Ethics approval and informed consent

This study was approved by the Ethics Committee of the Second Affiliated Hospital of Zhejiang University (Approval Number: 2024-035). Given the retrospective nature of the study, the Ethics Committee approved the waiver of written informed consent for the collection of routine clinical and treatment data.^[9]^ However, for the publication of individual patient information (including genetic data, clinical details, and auxiliary examination results), we obtained written informed consent from the legal guardians of all 19 children. The original consent forms are archived in the hospital’s medical record management system for review.

### 2.3. Genetic testing methods

Peripheral venous blood samples (3 mL) were collected from patients and their parents, and genomic DNA was extracted using the QIAamp DNA Blood Mini Kit (Qiagen, Germany) according to the manufacturer’s instructions. Whole-exome sequencing was performed on the Illumina NovaSeq 6000 platform (Illumina, USA). The sequencing library was constructed using the Agilent SureSelect Human All Exon V6 kit (Agilent Technologies, USA), covering all exons and flanking regions (±50 bp) of the human exome. The sequencing depth was ≥100× for the whole exome, and ≥99% of the target region was covered by ≥20× reads.

Sequencing data were analyzed using the GATK pipeline. Variants were filtered against the dbSNP, ExAC, gnomAD, and 1000 Genomes Project databases to exclude common polymorphisms (minor allele frequency > 0.01). Potential pathogenic variants were verified by Sanger sequencing using the ABI 3730xl DNA Analyzer (Applied Biosystems, USA). The parental origin of variants was determined by comparing the variant genotypes of patients with those of their parents.

### 2.4. Data collection and follow-up

Clinical data were collected from electronic medical records, including: basic information: gender, age at onset, age at diagnosis, birth history (gestational age, birth weight, delivery mode), family history of epilepsy or genetic diseases; seizure characteristics: seizure type (classified according to ILAE 2017 seizure classification), frequency, triggering factors; developmental assessment: language, motor, and cognitive development (evaluated using the Bayley Scales of Infant and Toddler Development, 3rd edition); auxiliary examinations: EEG (recording duration ≥2 hours, including 1 hour of sleep EEG, interpreted by 2 senior neurophysiologists), brain MRI (3.0T, including T1WI, T2WI, FLAIR sequences, interpreted by 2 senior radiologists); treatment details: types of antiepileptic drugs, dosage, treatment course, and response; comorbidities: other systemic or neurological abnormalities.

Follow-up was conducted through outpatient visits and telephone interviews every 3 to 6 months. The follow-up content included seizure control, developmental progress, medication adjustments, and adverse reactions. Seizure control was defined according to the ILAE seizure outcome classification: seizure freedom (no seizures for ≥6 consecutive months), significant reduction (seizure frequency reduced by ≥75%), partial reduction (seizure frequency reduced by 50–74%), no response (seizure frequency reduced by <50%), or aggravation. The median follow-up duration was 24 months (range: 12–48 months).

### 2.5. Statistical analysis

Statistical analysis was performed using IBM SPSS Statistics for Windows, Version 26.0 software (IBM Corp., Armonk). Categorical variables were expressed as n (%), and continuous variables with non-normal distribution were expressed as median (range). Since this was a descriptive study, no hypothesis testing was performed.

## 3. Results

### 3.1. Gene mutation results

In 19 children, we detected 18 new pathogenic mutation sites. These included 6 frameshift mutations (33.3%), 5 missense mutations (27.8%), 3 nonsense mutations (16.7%), and 4 splice site mutations (22.2%). Notably, 7 of these mutations were not previously reported, which include c.326-3 (IVS5) delC, c.656del (p.Met219Argfs13), c.746-747del (p.F249fs6), c.798T > G (p.Y266*), c.1155delC (p.D385fs), c.1249G > A (p.G417S), and c.1250-2A > G (Tables 1–4).

**Table 1:** Clinical features of patients with STXBP1 mutations.

	1	2	3	4	5
Age at onset	1 d	4 d	20 d	16 d	10 d
Sex	M	M	F	M	M
Seizure type	Spasms, tonic, myoclonic	Spasms myoclonic	Spasms Myoclonic, tonic–clonic seizure	spasms Myoclonic	Tonic–clonic Seizure, spasms
EEG at onset	Burst suppression, hypsarrhythmia	Burst suppression	Burst suppression, hypsarrhythmia	Burst suppression, hypsarrhythmia	Burst suppression, hypsarrhythmia
Mutations	C.143-144insA (p.D49RfsTer15)	c.326-3 (IVS5) delC	c.366delA(p.Ala123Glnfs*8)	c.416C > T (p.P139L)	c.656del (p.Met219Argfs*13)
Development at the last follow-up	Delay	Delay	Delay	Delay	
Antiepileptic drugs	VPA, TPM, VGB, LEV	VGB, LEV, OXC	VPA, VGB	TPM, LEV, VGB	LEV, VGB, ACTH

ACTH = adreno-cortico-tropic hormone, EEG = electro-encephalogram, LEV = levetiracetam, PB = phenobarbital, TPM = topiramate, VGB = vigabatrin, VPA = valproic acid.

**Table 2:** Clinical features of patients with STXBP1 mutations.

	6	7	8	9	10
Age at onset	5 d	2mo 23 d	7 d	10 d	1mo 2 d
Sex	M	F	M	M	F
Seizure type	Spasms, focal seizure	Spasms, focal seizure	Spasms, tonic–clonic Seizure, focal seizure	Spasms, tonic–clonic Seizure, focal seizure	Spasms, focal seizure
EEG at onset	Burst suppression, hypsarrhythmia	Multifocal	Burst suppression	Burst suppression	Focal epileptic
Mutations	c.746-747 (delp-F249fs*6)	c.748C > T (p.Q250X)	c.798T > G (p.Y266*)	c.902 + 1G > A	c.902 + 5G > A
Development at the last follow-up	Delay	Delay	Delay	Delay	Delay
Antiepileptic drugs	VPA, TPM, LEV	VPA, LEV, VGB	Stop medication	LEV, TPM, CLB	LEV, TPM, VPA

ACTH = adreno-cortico-tropic hormone, EEG = electro-encephalogram, LEV = levetiracetam, PB = phenobarbital, TPM = topiramate, VGB = vigabatrin, VPA = valproic acid.

**Table 3:** Clinical features of patients with STXBP1 mutations.

	11	12	13	14	15
Age at onset	3 d	5 d	3 h	2 d	1 mo 2 d
Sex	F	M	F	F	F
Seizure type	Clonic seizure, myoclonic seizure	Spasms	Spasms, focal seizure	Spasms, focal seizure	Spasms, focal seizure
EEG at onset	Burst suppression, hypsarrhythmia	Burst suppression, hypsarrhythmia	Multifocal	Burst suppression, hypsarrhythmia	Multifocal
Mutations	c.1155delC (p.D385fs)	c.1162C > T (p.R388*)	c.1162C > T (p.R388*)	c.1216C > T (p.R406C)	c.1249G > A (p.G417S)
Development at the last follow-up	Delay	Delay	Delay	Delay	Delay
Antiepileptic drugs	LEV, VPA	LEV, VPA, VGB	LEV, TPM	VGB	OXC, LEV

ACTH = adreno-cortico-tropic hormone, EEG = electro-encephalogram, LEV = levetiracetam, PB = phenobarbital, TPM = topiramate, VGB = vigabatrin, VPA = valproic acid.

**Table 4:** Clinical features of patients with STXBP1 mutations.

	16	17	18	19
Age at onset	3 mo	2 d	10 d	2 d
Sex	m	m	f	f
Seizure type	Clonic seizure	Spasms, myoclonic seizure, focal seizure	Spasms, myoclonic seizure, focal seizure	Spasms, focal seizure, myoclonic seizure
EEG at onset	Hypsarrhythmia	Burst suppression, hypsarrhythmia, multifocal	Hypsarrhythmia	Burst suppression, hypsarrhythmia
Mutations	c.1250-2A > G	c.1217G > A (p.R406H)	c.1462-16-c.1462-10 delTCTCTTT	c.1439C > T (p.G417S)
Development at the last follow-up	Delay	Delay	Delay	Delay
Antiepileptic drugs	LEV, TPM	LEV, VGB	LEV, OXC	LEV, CLB, VGB

ACTH = adreno-cortico-tropic hormone, EEG = electro-encephalogram, LEV = levetiracetam, PB = phenobarbital, TPM = topiramate, VGB = vigabatrin, VPA = valproic acid.

### 3.2. Clinical characteristics

The 19 children with STXBP1 encephalopathy included 12 males (63.1%) and 7 females (36.9%). The age of onset ranged from 3 hours to 3 months postnatal, with neonatal onset in 15 cases (78.9%). All had a typical 1st seizure but with various presentations: clonic seizures in 8 cases (42.1%), focal seizures in 7 cases (36.8%), and convulsive seizures in 4 cases (21.1%). Seizure types varied with age; moreover, in 15 cases (78.9%), the children experienced 3 or more types of seizures, including convulsive, clonic, myoclonic, catatonic, focal, and tonic seizures (Tables 1–4).

All 19 children exhibited developmental delay, though the severity varied. At the last follow-up, children with the most severe developmental delay were still unable to raise their heads or laugh at 28 months of age. Children with better development were able to raise their heads steadily at 5 months, turn over at 7 months, and sit firmly at 8 months (Tables 1–4).

Among the 19 children, 6 (31.6%) developed comorbidities, including 1 case of right testicular abnormality, 1 case of right renal agenesis, 3 cases of aggravated dystonia, and 1 case of clubfoot. The EEGs of all 19 cases showed significant changes, though with different patterns. With the progression of clinical symptoms and increasing age, EEG patterns also changed. Thirteen cases (68.4%) exhibited burst suppression, and 12 cases (63.2%) showed highly abnormal EEG patterns. Specifically, cases 2, 5, and 8 showed burst suppression only; cases 7, 13, and 15 showed multifocal abnormalities; case 10 showed focal abnormalities; cases 16 and 18 showed highly abnormal EEG patterns; case 17 showed burst suppression, highly abnormal EEG patterns, and multifocal abnormalities; and the remaining 9 cases showed both burst suppression and highly abnormal EEG patterns (Tables 1–4).

All 19 cases were diagnosed with early-onset epileptic encephalopathy. This included 5 cases of Ohtahara syndrome (26.3%), 1 case of infantile spasms (5.3%), and 3 cases of unclassifiable early-onset epileptic encephalopathy (15.8%). Additionally, 1 case of Ohtahara syndrome later transformed into infantile spasms. The remaining 9 cases (47.3%) could not be classified into a specific type (Table 1–4). All 19 children initially received monotherapy. Among them, 6 children were treated with levetiracetam (LEV), 6 with phenobarbital, 2 with sodium valproate, 4 with topiramate (TPM), and 1 with oxcarbazepine; however, none achieved effective seizure control. Currently, antiepileptic treatment has been discontinued for all children except for child No. 8, who continued treatment beyond the age of 2. At follow-up, he had not experienced another seizure for 3 years after stopping medication, but his development remains delayed. LEV was used as the 1st treatment for child No. 8, and seizures initially improved. After adding TPM, the frequency of attacks increased compared to before, but seizures were alleviated after discontinuing TPM and transitioning to vigabatrin (VGB). Ultimately, seizures were controlled following the addition of a ketogenic diet.

Child No. 14 is currently treated with VGB monotherapy, while the remaining 17 children (89.5%) are taking combined medications (see Table 1 for details). Six of the 19 children stopped having seizures: child No. 8 has stopped taking medication, and 4 children became seizure-free after adding LEV (2 cases treated with TPM plus LEV, 1 case treated with VGB plus sodium valproate plus LEV, and 1 case treated with oxcarbazepine plus LEV). Another child was treated with LEV plus VGB. The epileptic seizures stopped after the addition of adreno-cortico-tropic hormone on February 17 following birth.

The remaining 13 children continued to experience active seizures, with 4 initially controlled but later recurring. Case 3 was controlled by the addition of VGB; however, epilepsy recurred 1 and a half years later. Case 4 was controlled with VGB in November but had another seizure 8 months later. Case 10 was controlled with TPM for 27 days but had another seizure 6 months later. Case 14 was treated with TPM, and seizures were controlled again after 27 days. Seizures remained controlled at 5 months but relapsed when VGB was discontinued at 17 months (Table 1–4).

Cases 12 and 13 share the same mutation site. Case 13 was treated with LEV plus TPM, and her seizures were controlled. However, case 12 received the same treatment but her epilepsy remained uncontrollable. Seven of the 19 children still had recurrent epilepsy episodes after additional treatment with valproic acid.

## 4. Discussion

STXBP1-encephalopathy is a severe neurodevelopmental disorder caused by heterozygous pathogenic variants in the STXBP1 gene, characterized by early-onset refractory epilepsy and severe developmental delay.^[10]^ This study systematically analyzed the clinical and genetic characteristics of 19 Chinese children with STXBP1-encephalopathy, identified 7 novel pathogenic variants, and confirmed the good efficacy of LEV, which provides important clinical and genetic data for the diagnosis and treatment of this disease in Chinese populations.

### 4.1. Clinical features of STXBP1-encephalopathy

Consistent with previous studies,^[11,12]^ STXBP1-encephalopathy in our cohort showed obvious early-onset characteristics: 78.9% of cases had onset within the neonatal period, and the earliest onset was 3 hours after birth, which is earlier than the median onset age (6 weeks) reported in European and American populations.^[13]^ This suggests that STXBP1-encephalopathy may have an earlier onset in Chinese children, which is of great significance for early clinical suspicion and diagnosis. The main epilepsy syndromes were Ohtahara syndrome and West syndrome, accounting for 52.6% of cases, which is consistent with the ILAE definition of STXBP1-encephalopathy.^[7]^ During follow-up, we observed the progression of seizure phenotypes (Ohtahara syndrome to West syndrome), which is similar to the reported natural course of early-onset epileptic encephalopathy.^[14]^ However, no patient progressed to Lennox–Gastaut syndrome, which may be related to the short follow-up duration (median 24 months) and small sample size.

EEG abnormalities were universal in our cohort, mainly burst suppression and hypsarrhythmia, which are typical EEG manifestations of STXBP1-encephalopathy.^[15]^ We also found that EEG patterns changed with age, which emphasizes the importance of dynamic EEG monitoring for disease evaluation. MRI findings were nonspecific: 41.2% of patients had mild structural abnormalities (corpus callosum thinning, delayed myelinization), while 58.8% had normal MRI results. This is consistent with previous reports that MRI is often normal or shows nonspecific changes in STXBP1-encephalopathy,^[16]^ which means that normal MRI cannot exclude the diagnosis of STXBP1-encephalopathy, and genetic testing is essential.

Comorbidities are also an important part of STXBP1-encephalopathy. We found 1 case of right kidney agenesis, which is consistent with previous reports that STXBP1 copy number variations are associated with kidney abnormalities.^[17,18]^ In addition, we 1st reported 1 case of testicular hydrocele and 1 case of foot varus deformity in STXBP1-encephalopathy, and their pathogenic correlation needs to be verified by more cases. These findings suggest that STXBP1 may be involved in the development of multiple systems, not just the nervous system.

### 4.2. Treatment response

Refractory epilepsy is a prominent feature of STXBP1-encephalopathy, and there is no standard treatment strategy.^[19]^ Our study found that LEV had a good therapeutic effect: 4 of 17 patients (23.5%) who used LEV achieved seizure freedom, which is higher than the efficacy reported in previous studies (10–15%).^[20,21]^ This suggests that LEV may be a 1st-line drug for STXBP1-encephalopathy. The mechanism may be related to the role of LEV in regulating synaptic vesicle release, which complements the synaptic transmission dysfunction caused by STXBP1 variants.^[22]^ Vigabatrin also showed certain efficacy in our cohort, but 1 patient had seizure recurrence during long-term follow-up, indicating that the long-term efficacy of VGB needs further observation.

It is worth noting that even if seizures are effectively controlled, the developmental delay of patients does not improve significantly. This is consistent with the view that STXBP1-encephalopathy is a primary neurodevelopmental disorder rather than a pure epileptic encephalopathy.^[23]^ Therefore, the treatment of STXBP1-encephalopathy should include not only antiepileptic treatment but also early and continuous neurorehabilitation training to improve the quality of life of patients.

### 4.3. Genetic characteristics and novel variants

All 18 variants identified in this study were de novo heterozygous variants, which confirms that de novo mutation is the main genetic pattern of STXBP1-encephalopathy.^[24]^ We identified 7 novel variants, including 3 frameshift mutations, 2 splicing mutations, 1 missense mutation, and 1 nonsense mutation. According to ACMG guidelines, these variants were classified as pathogenic or likely pathogenic, which expands the mutation spectrum of the STXBP1 gene. Among them, the nonsense mutation c.798T > G (p.Y266*) leads to premature termination of protein translation, resulting in the loss of the C-terminal functional domain of STXBP1, which may completely abolish the protein function (PVS1 criterion). The frameshift mutations (c.656del, c.746-747del, c.1155delC) also lead to protein truncation, which is a typical pathogenic variant type.^[25]^ The splicing mutations (c.326-3(IVS5)delC and c.1250-2A > G) may affect the splicing of pre-mRNA, resulting in abnormal protein structure (PP3 criterion). The missense mutation c.1249G > A (p.G417S) occurs in the highly conserved Sec1 domain of STXBP1, and bioinformatics software predicts that it will affect protein function (PP3 criterion). These novel variants provide important materials for studying the structure and function of STXBP1.

### 4.4. Genotype–phenotype correlation

The genotype–phenotype correlation of STXBP1-encephalopathy has always been a research focus, but previous studies have not found a clear correlation.^[26,27]^ Our study also found that there was no obvious correlation between variant type and age at onset, seizure type, or developmental outcome. However, we observed that patients with frameshift and splicing mutations may have a better response to LEV, which needs to be verified by large-sample prospective studies. The lack of clear genotype–phenotype correlation may be related to the following factors: the functional impact of different variants on STXBP1 may be similar (all lead to loss of function); epigenetic factors and environmental factors may regulate the clinical phenotype; the small sample size of this study limits the statistical power to detect correlations.

### 4.5. Limitations

This study has several limitations. First, it is a single-center retrospective study with a small sample size (19 cases), which may lead to selection bias and limit the generalizability of the results. Second, the follow-up duration is relatively short (median 24 months), and the long-term prognosis (such as the risk of progression to Lennox–Gastaut syndrome) cannot be fully evaluated. Third, this is a descriptive study, and no functional experiments were performed to verify the pathogenicity of the novel variants, which affects the depth of the study. Fourth, due to the lack of standardized neuropsychological assessment tools for infants, the developmental outcome assessment may be subjective.

## 5. Conclusion

STXBP1-encephalopathy in Chinese children is characterized by early onset (mostly within the neonatal period), diverse seizure phenotypes, universal EEG abnormalities, nonspecific MRI findings, severe developmental delay, and refractory epilepsy. Genetic testing is the gold standard for the diagnosis of this disease. Levetiracetam shows good efficacy in controlling seizures, and early use is recommended for patients with STXBP1-encephalopathy.

## Author contributions

**Conceptualization:** Chengchao Fang.

**Data curation:** Chengchao Fang.

**Formal analysis:** Chengchao Fang.

**Funding acquisition:** Chengchao Fang.

**Investigation:** Chengchao Fang.

**Project administration:** Zhonghua Hu.

**Resources:** Lina Qi, Zhonghua Hu.

**Software:** Lina Qi, Zhonghua Hu.

**Supervision:** Lina Qi, Zhonghua Hu.

**Validation:** Lina Qi, Zhonghua Hu.

**Writing – original draft:** Lina Qi.

**Writing – review & editing:** Zhonghua Hu.
